# The Viloxazine Paradox: A Noradrenergic Agent's Journey From Antidepressant Obscurity to ADHD Precision Therapy

**DOI:** 10.1002/cns.70839

**Published:** 2026-04-07

**Authors:** Ghaith K. Mansour, Sabry Babiker H. Sayed, Sajjad Ghanim Al‐Badri, Mohammed Gamaleldin Abdulrahim Abdalla, Dina Essam Abo‐elnour

**Affiliations:** ^1^ Department of Pharmaceutical Sciences, College of Pharmacy Alfaisal University Riyadh Saudi Arabia; ^2^ Faulty of Medicine and Health Sciences International University of Africa Khartoum Sudan; ^3^ Clinical Skills Department, General Medicine Practice Program, Batterjee Medical College Dammam Saudi Arabia; ^4^ College of Medicine University of Warith Al‐Anbiyaa Karbala Iraq; ^5^ College of Medicine University of Baghdad Baghdad Iraq; ^6^ Faculty Medicine and Health Sciences International University of Africa Khartoum Sudan; ^7^ Faculty of Medicine Zagazig University Zagazig Egypt

**Keywords:** ADHD, serotonin‐norepinephrine modulatory agent (SNMA), viloxazine

## Abstract

**Aims:**

Viloxazine is a serotonin–norepinephrine modulatory agent that was recently approved by the U.S. Food and Drug Administration for the treatment of attention‐deficit/hyperactivity disorder (ADHD) in children, adolescents, and adults. This review aims to summarize the regulatory history, pharmacology, pharmacokinetics, clinical efficacy, safety, and current therapeutic role of viloxazine, while also highlighting gaps in the evidence and future research directions.

**Methods:**

A narrative literature review was conducted using PubMed, Google Scholar, and FDA regulatory documents. Relevant peer‐reviewed articles, including clinical trials, reviews, meta‐analyses, and prescribing information, were screened for data on viloxazine's historical antidepressant use, mechanism of action, pharmacokinetics, efficacy in ADHD, safety profile, drug interactions, and comparative effectiveness. Non‐peer‐reviewed sources were excluded.

**Results:**

Viloxazine was originally marketed in Europe in the 1970s as an antidepressant and showed a favorable safety profile over decades of use. Its pharmacological profile includes moderate norepinephrine reuptake inhibition with additional serotonergic activity, particularly 5‐HT2C agonism and 5‐HT2B antagonism, which supported its repurposing as an extended‐release formulation for ADHD. Multiple randomized, placebo‐controlled trials demonstrated that viloxazine extended‐release significantly improves ADHD symptoms in pediatric, adolescent, and adult populations. Reported effect sizes were generally smaller than those of stimulants but comparable to atomoxetine. The most common adverse events were somnolence, headache, nausea, decreased appetite, and insomnia. Viloxazine also has a pharmacokinetic profile compatible with once‐daily dosing, although clinically relevant drug interactions may occur through CYP1A2 inhibition.

**Conclusion:**

Viloxazine expands the range of non‐stimulant treatment options for ADHD and represents a successful example of drug repurposing in neuropsychiatry. Current evidence supports its efficacy and acceptable tolerability across the lifespan, particularly for patients who do not tolerate or respond adequately to stimulants. Nevertheless, evidence for its use beyond ADHD remains limited, and future studies should focus on head‐to‐head comparisons with other ADHD therapies, long‐term safety and efficacy, predictors of response, and potential off‐label psychiatric applications.

## Graphical abstract

Viloxazine, a serotonin–norepinephrine modulating agent, has been repurposed from an antidepressant to a novel non‐stimulant for ADHD. Its multimodal mechanism and Phase II/III trials demonstrate clinically meaningful symptom reduction with a distinct safety and interaction profile, offering an alternative for patients who cannot tolerate stimulants.

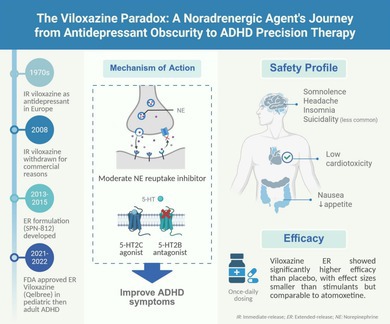

## 1 Introduction

Viloxazine is a novel serotonin‐norepinephrine modulatory agent (SNMA) recently approved by the FDA for ADHD across the lifespan [1]. It was first synthesized and marketed in 1974 as an immediate‐release antidepressant (brand names Vivalan, Emovit) in Europe [2]. Over three decades of European use yielded a favorable safety profile (notably low cardiotoxicity vs. TCAs) [3]. The historical experience in depression and its unique pharmacology prompted development of an extended‐release (ER) formulation (SPN‐812) for ADHD. In April 2021, the FDA approved Qelbree (viloxazine ER) for pediatric ADHD (6–17 years), and in May 2022 for adult ADHD [1]. This review synthesizes peer‐reviewed evidence and official labels to provide a comprehensive overview of viloxazine's pharmacology, clinical uses (current and historical), trial data, safety/tolerability, drug interactions, comparative efficacy, and regulatory milestones. We include FDA‐approved indications (pediatric and adult ADHD), historical antidepressant use, and any off‐label considerations. Methodology and evidence quality are appraised to inform clinicians and researchers on viloxazine's role in neuropsychiatry.

## 2 Methodology

A literature search was conducted on PubMed, Google Scholar, and FDA databases using terms “viloxazine,” “viloxazine extended‐release,” “SPN‐812,” “Qelbree,” “viloxazine ADHD,” and “viloxazine depression.” We included peer‐reviewed articles (clinical trials, reviews, meta‐analyses) and official FDA labeling. Non–peer‐reviewed sources (press releases, news articles, blogs) were excluded. FDA prescribing information and regulatory documents were consulted for approved indications, dosing, pharmacokinetics, and warnings [4]. Key clinical trials (Phase II/III RCTs, open‐label extensions) were identified and data extracted for efficacy endpoints (ADHD rating scales, CGI) and safety outcomes. Trial quality, bias risk, and evidence certainty were evaluated (recognizing most trials are industry‐sponsored) to provide critical appraisal. Where data were sparse (e.g., historical antidepressant studies), we relied on reviews and unpublished reports referenced by regulatory sources [2, 4]. The review synthesizes findings narratively, structured by the specified sections.

## 3 Regulatory History and Development Timeline

Viloxazine was first synthesized in the 1970s and approved as an immediate‐release (IR) antidepressant in the United Kingdom in 1974, followed by marketing in several European countries where it remained available for approximately 30 years [3]. During the 1990s and 2000s, viloxazine continued to be used in Europe without major safety concerns. However, by 2008, the IR formulation was withdrawn globally due to commercial reasons rather than safety‐related issues, and it had never been submitted for U.S. approval [4].

Between 2013 and 2015, Supernus Pharmaceuticals initiated the repurposing of viloxazine, developing an extended‐release (ER) formulation known as SPN‐812. This strategic pivot was based on the compound's norepinephrine reuptake inhibition (NRI) properties, which suggested therapeutic potential in attention‐deficit/hyperactivity disorder (ADHD). Early Phase II studies targeting pediatric populations commenced during this period. From 2016 to 2020, Supernus executed a comprehensive clinical development program comprising multiple Phase II and III trials. Key studies included a pivotal Phase II pediatric trial by Johnson et al., followed by two Phase III pediatric trials, an adolescent Phase III trial, and a Phase III adult study. Cardiac safety was addressed through a rigorous thorough QTc study published confirming the absence of QT prolongation risk [4]. On April 26, 2021, the U.S. Food and Drug Administration (FDA) approved viloxazine ER under the brand name Qelbree for the treatment of ADHD in children and adolescents aged 6–17, marking it as the first novel non‐stimulant ADHD medication to receive FDA priority review and approval in this population [4]. Subsequently, on July 8, 2021, New Drug Application (NDA) 211,964 was officially approved, and the U.S. Prescribing Information was published. The therapeutic indication was later expanded: on May 2, 2022, the FDA approved Qelbree for adults aged 18 and older, making it the first novel non‐stimulant for adult ADHD in over two decades, as highlighted by *PharmacyTimes*; the product label was updated in April 2022 to reflect this change [4].

From 2022 through 2024, several clinical investigations remain ongoing, including long‐term safety assessments through open‐label extension trials and studies exploring viloxazine in combination with stimulant medications or for off‐label applications such as substance use disorders. Although viloxazine is currently only approved for ADHD, the drug maintains U.S. patent exclusivity into the late 2020s, securing market protection. No regulatory filings have yet been pursued for non‐ADHD indications.

In summary, viloxazine's regulatory journey reflects a repurposing success story: a once‐abandoned antidepressant reintroduced as a pediatric ADHD therapy, followed by rapid expansion to adults. FDA reviews (NDA documents) note the 2008 withdrawal due to business decisions [4]. The recent approvals were supported by substantial clinical data and a favorable safety profile.

## 4 Pharmacology and Mechanism of Action

**FIGURE 1:**
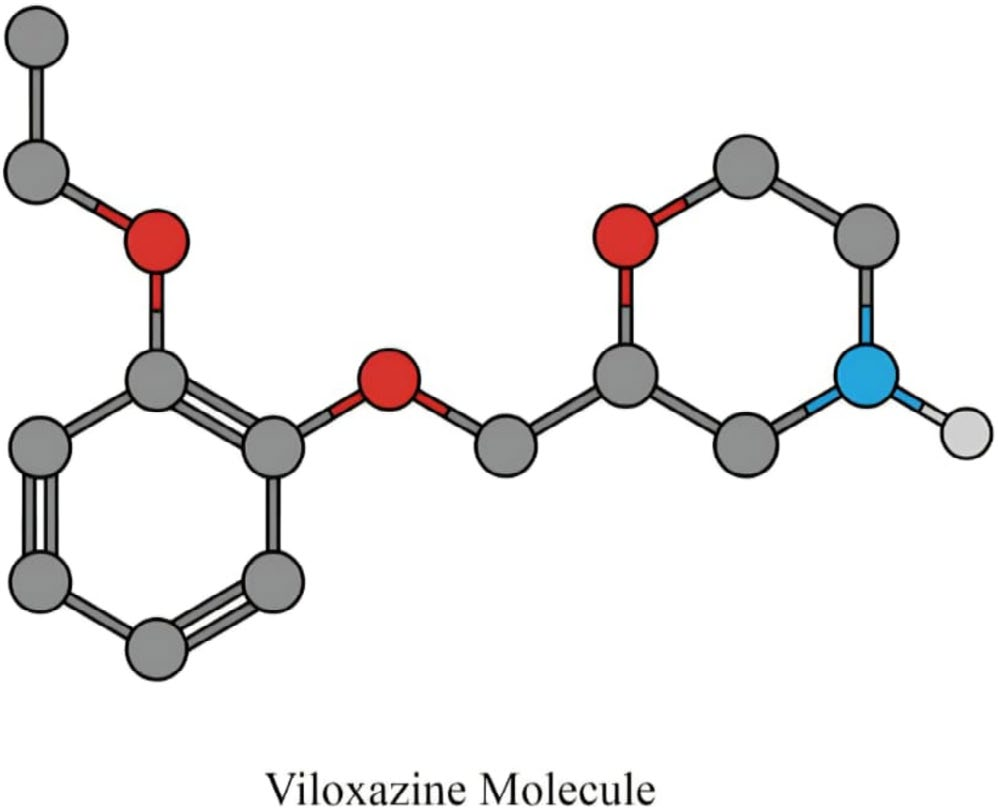
Chemical structure of viloxazine. Viloxazine is a bicyclic serotonin–norepinephrine modulating agent (SNMA) with a distinct structural scaffold. Its pharmacological activity is derived from moderate norepinephrine transporter (NET) inhibition combined with serotonergic receptor modulation, including 5‐HT2C agonism and 5‐HT2B antagonism. This multimodal profile underlies its clinical efficacy in attention‐deficit/hyperactivity disorder (ADHD).

**FIGURE 2:**
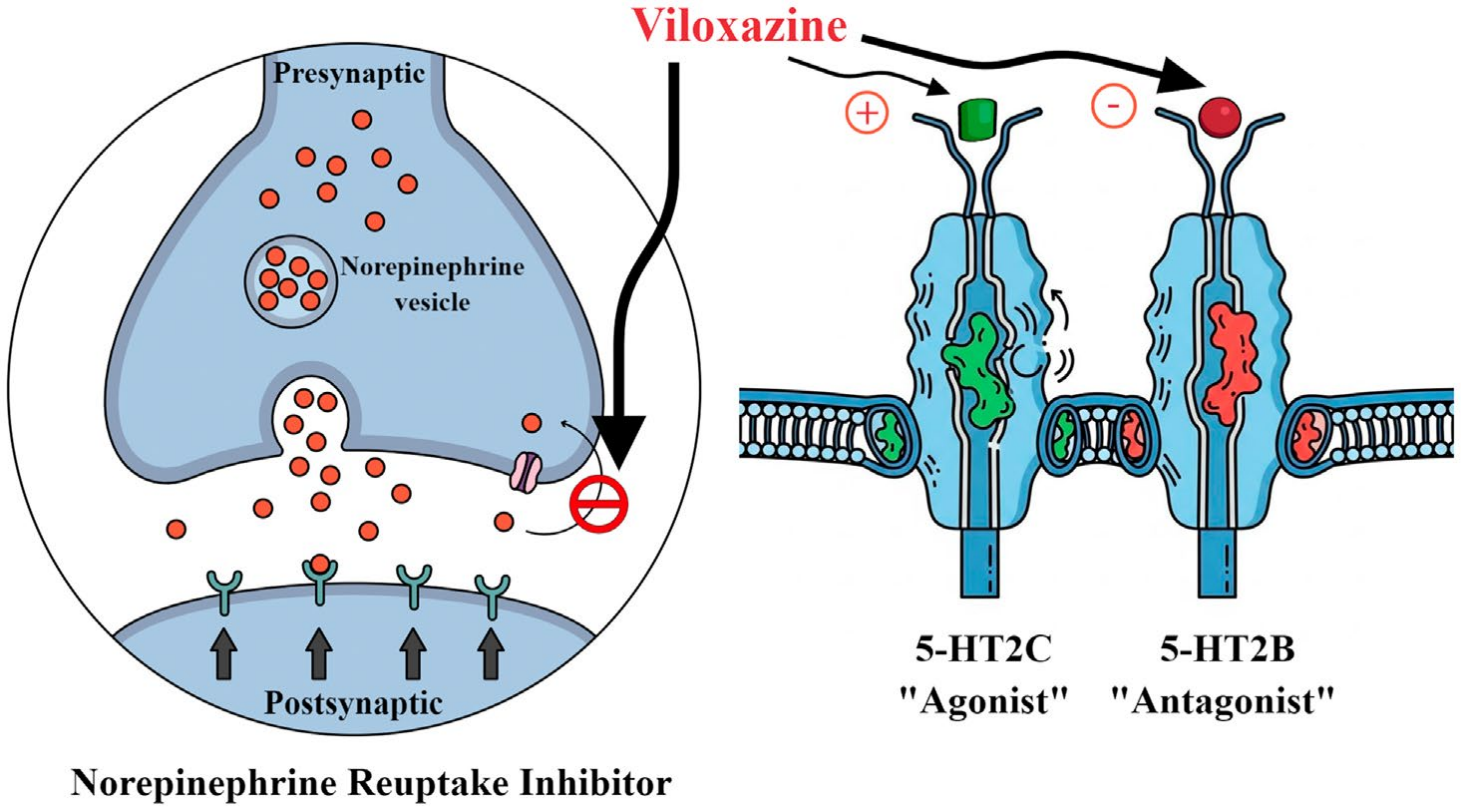
Mechanism of action of viloxazine. Viloxazine functions as a serotonin–norepinephrine modulating agent (SNMA). It moderately inhibits the norepinephrine transporter (NET), leading to increased norepinephrine availability in the prefrontal cortex. In addition, it acts as an agonist at the 5‐HT2C receptor, enhancing cortical serotonin activity, and as an antagonist at the 5‐HT2B receptor, potentially reducing valvulopathy risk while contributing to serotonergic modulation. This multimodal mechanism underlies viloxazine's therapeutic effects in attention‐deficit/hyperactivity disorder (ADHD).

Viloxazine's pharmacology is complex and distinct from typical stimulants (Table 1). It was historically classified as a selective NRI based on animal and human studies [2]. Early work showed viloxazine potentiates noradrenergic effects in multiple models [2], albeit with moderate potency: its NE uptake K_i_ (~2300 nM) is much weaker than atomoxetine or reboxetine [2]. Viloxazine has negligible activity at dopamine transporters (DAT K_D_ > 100,000 nM) [2]. Recent preclinical research reveals important serotonergic actions. Viloxazine is an antagonist at 5‐HT_2B_ receptors and agonist at 5‐HT_2C_. Emerging evidence suggests that serotonin (5‐HT) plays an important modulatory role in attention, impulsivity, and emotional regulation—core domains affected in ADHD. Serotonergic projections from the dorsal raphe nucleus to the prefrontal cortex and striatum can influence dopaminergic and noradrenergic activity, thereby modulating executive and motivational control. Altered 5‐HT signaling, particularly involving 5‐HT2A and 5‐HT2C receptors, has been associated with impulsivity and hyperactivity in both animal and human studies. Consequently, agents that enhance cortical serotonin transmission or selectively activate 5‐HT2C receptors may improve inhibitory control and attentional stability. Viloxazine's 5‐HT2C agonism and 5‐HT2B antagonism are thought to contribute to its therapeutic profile by restoring serotonergic balance and modulating downstream catecholaminergic pathways relevant to ADHD symptomatology [2] (Figures 1 and 2, Table 1). These actions are predicted to have high receptor occupancy at clinical doses [2]. In vivo rodent microdialysis, viloxazine increased extracellular 5‐HT and NE in prefrontal cortex (PFC) [2]. The increase in PFC 5‐HT is noteworthy, given PFC's role in attention and executive function. The JEP study concludes that viloxazine should be considered a serotonin‐norepinephrine modulating agent (SNMA), with 5‐HT modulation being a dominant component of its effect [2] This may differentiate viloxazine from pure NRIs like atomoxetine (see Table 1 for the receptor/target profile).

**TABLE 1:** Pharmacodynamic targets and receptor profile of viloxazine.

Target receptor/transporter	Action type	Functional outcome	Relative affinity
Norepinephrine Transporter (NET)	Inhibition	↑ Prefrontal NE	Moderate (Ki ~2300 nM)
5‐HT2C Receptor	Agonist	↓ Appetite, ↑ PFC 5‐HT (attention, cognition)	High
5‐HT2B Receptor	Antagonist	May reduce risk of valvulopathy, ↑ PFC 5‐HT	High
Dopamine Transporter (DAT)	Negligible	No addiction potential	Very low (KD > 100,000 nM)
Histamine, Muscarinic Receptors	None	No sedation or anticholinergic effects	None

Viloxazine also shows modest effects on other neurotransmitters. It has little affinity for cholinergic or histaminergic receptors [2], unlike many TCAs. It is a very weak MAO inhibitor [3]. Early animal data indicated some dopaminergic enhancement in PFC (via NET inhibition), which may contribute to attention effects [3]. Although viloxazine shows negligible affinity for dopamine transporters, dopamine clearance in the prefrontal cortex is primarily mediated by the norepinephrine transporter (NET). Consequently, inhibition of NET reduces dopamine reuptake in this region, leading to elevated extracellular dopamine despite minimal direct DAT binding. Preclinical microdialysis studies in rats have confirmed that NET inhibitors, including viloxazine, enhance cortical dopamine availability through this indirect mechanism, contributing to improved attention and executive functioning [2, 3]. However, viloxazine's low DAT affinity and minimal nucleus accumbens dopamine release suggest a low abuse liability [3]. Clinically, viloxazine exhibits stimulant‐like benefits without amphetamine‐like properties [5]. In summary, viloxazine's mechanism combines moderate NE reuptake inhibition with 5‐HT_2C_ agonism and 5‐HT_2B_ antagonism, yielding indirect increases in cortical 5‐HT and NE [2]. This multimodal profile underlies its therapeutic effects in ADHD and likely contributed to its antidepressant action.

## 5 Pharmacokinetics and Pharmacodynamics

Viloxazine ER is well‐absorbed, with peak plasma levels ~5 h post‐dose (T_max_ ≈5 h) (Table 2) [4]. Its oral bioavailability is high (~88%) [1]. Viloxazine's elimination half‐life is approximately 7.0 h [4], supporting once‐daily dosing. Steady‐state is reached within 2–3 days, with minimal accumulation observed [1]. The drug is moderately bound to plasma proteins (76%–82%) [4], similar in children and adults. Metabolism is primarily hepatic via CYP2D6, UGT1A9, and UGT2B15 [1]. The major metabolite is 5‐hydroxyviloxazine‐glucuronide [1]. Co‐administration studies indicate that viloxazine ER potently inhibits CYP1A2 (likely mechanism‐based) and moderately inhibits CYP2D6 and CYP3A4 [1]. This can increase exposure of substrates (e.g., caffeine, clozapine, warfarin) [1], Renal excretion accounts for the majority (~90%) of dose recovery, mostly as glucuronide metabolites [1]. No clinically significant food effect was observed. Pediatrics show higher plasma levels (130%–250% of adult AUC at equivalent dose) [1], necessitating age‐based dosing. In patients with hepatic or renal impairment, exposure increases (by ~1.5‐fold in moderate liver disease; up to 2.5‐fold in severe kidney disease), so dosage adjustments are recommended (Table 2) [1].

**TABLE 2:** Pharmacokinetics of viloxazine ER (Qelbree).

Parameter	Value	Clinical relevance
Bioavailability	~88%	High oral absorption
Tmax	~5 h	Suitable for once‐daily dosing
Half‐life (t½)	~7 h	Steady‐state in ~2–3 days
Protein Binding	76%–82%	Moderate; similar in adults and children
Metabolism	CYP2D6, UGT1A9/2B15	Drug–drug interaction risk (CYP1A2 inhibition)
Major Excretion Route	Renal (mostly glucuronides)	Requires dose adjustment in renal disease
Pediatric AUC vs. Adult	↑130%–250%	Lower doses needed in children

Pharmacodynamically, viloxazine ER does not prolong cardiac QTc or QRS intervals [6]. A thorough QT study confirmed no proarrhythmic effects at supratherapeutic doses [6]. Blood pressure and heart rate may increase slightly (mean SBP/DBP by ~1.5/1.4 mmHg), but these changes are generally not clinically meaningful [6]. Viloxazine's lack of anticholinergic or antihistaminergic effects is reflected in its tolerability profile (no sedation via those pathways) [2]. In sum, viloxazine ER's PK/PD profile supports once‐daily dosing with rapid absorption, moderate half‐life, and dose‐proportional exposure (see Table 2).

## 6 Clinical Trials and Indications

Viloxazine ER has been rigorously tested in pediatric ADHD (Table 3 provides a trial overview). A pivotal Phase II trial (*n* ≈100) in 6–12‐year‐olds demonstrated dose‐dependent improvement in ADHD‐Rating Scale (ADHD‐RS) and CGI scores [6]. Based on that, three Phase III RCTs were conducted. Nasser et al. randomized 477 children (6–11 years) to once‐daily viloxazine ER 100 mg or 200 mg or placebo for 6 weeks [7]. Both active doses produced significant ADHD‐RS‐5 total score reductions from baseline (mean change ~ −10 to −11) versus placebo (−7.0; *p* ≤ 0.0004) [7]. Clinical Global Impression of Improvement (CGI‐I) response rates were also higher with viloxazine (200 mg: 49%; placebo: 23%; *p* < 0.001) [7]. Treatment‐related adverse events (≥ 5%) were somnolence, decreased appetite, and headache [7]. Importantly, these trials found early onset of effect (significant by week 1) and a favorable tolerability profile compared to stimulants [6, 7].

**TABLE 3:** Summary of ADHD clinical trials for viloxazine ER.

Study (year)	Population	Duration	Dose(s) tested	Primary outcome	Result (vs placebo)
Nasser et al. (2020) [7]	Children (6–11)	6 weeks	100 mg, 200 mg	ADHD‐RS‐5	−10 to −11 pts. (*p* < 0.001)
Adolescents Phase III	Teens (12–17)	6 weeks	400 mg, 600 mg	ADHD‐RS‐5	−18.3 pts. (*p* = 0.008)
Nasser et al. (2022) [8]	Adults (18–65)	6 weeks	200–600 mg	AISRS total	−15.5 vs. −11.7 (*p* = 0.004)
Open‐label extension	Mixed Pop.	6–12 months	Maintenance	Symptom durability	Maintained improvement

An open‐label *phase II dose‐finding* study also tested 200, 300, 400 mg/day in children (registered as SPN‐812‐302). The 200 and 300 mg doses were effective, guiding the chosen pediatric dosing range (100–400 mg/day) [6]. A post hoc responder analysis indicated most responders achieved effect within 2 weeks, and no major safety issues emerged [9].

Adolescent trials mirrored the pediatric program. A Phase III RCT randomized 293 adolescents to viloxazine ER 400 mg, 600 mg, or placebo [10]. At study end, the 400 mg group showed a significantly greater mean reduction in ADHD‐RS‐5 total score (−18.3) than placebo (−13.2; *p* = 0.008) [10]; the 600 mg group improved (−16.7) but did not reach statistical significance (*p* = 0.071) [10]. The 400 mg dose also yielded higher CGI‐I response (44% vs. 33%, *p* = 0.042). Common adverse events included somnolence, fatigue, and decreased appetite. These results supported an adolescent dosage of 200–400 mg/day (as 400 mg was effective and better tolerated than 600 mg).

Two Phase III randomized, double‐blind, placebo‐controlled trials have evaluated viloxazine extended‐release (ER) for the treatment of adults with ADHD. The published trial by Nasser et al. (CNS Drugs 2022) enrolled 374 adults (mean age ~35) with moderate‐to‐severe ADHD [8]. Subjects were randomized 1:1 to viloxazine ER (200–600 mg/day titrated to optimal) or placebo for 6 weeks. The primary endpoint, change in Adult ADHD Investigator Symptom Rating Scale (AISRS) total score, favored viloxazine (−15.5 vs. −11.7; *p* = 0.004) [8]. Key secondary CGI‐Severity change was also significantly improved (viloxazine −1.4 vs. placebo −1.0; *p* = 0.0023) [8]. Statistically significant improvements were seen in both AISRS subscales (Inattention *p* = 0.0015; Hyperactivity *p* = 0.038) and in executive function measures (BRIEF‐A) [8]. Adverse events > 5% (viloxazine vs. placebo) included insomnia (not reported for placebo), headache (17% vs. 7%), somnolence (6% vs. 2%), nausea (12% vs. 3%), and decreased appetite (10% vs. 3%) [1]. Discontinuation due to adverse events was higher with viloxazine (17.6%, most due to insomnia and nausea) compared to placebo [11]. In summary, this trial demonstrated a statistically significant and clinically meaningful reduction in ADHD symptoms with viloxazine ER in adults. These data formed the basis for FDA adult ADHD approval (see Table 3 for primary outcomes across trials).

A second Phase III trial in adults was completed but results are unpublished; however, pooled data in the Qelbree label indicate replicable efficacy [1]. A long‐term open‐label extension in adults (*n* = 159) demonstrated sustained symptom improvement and tolerability over 6–12 months [11].

### 6.1 Historical Antidepressant Use (Adults)

Viloxazine was historically used as an antidepressant in adults. It was first licensed in the UK in 1974 and marketed in Europe for ~30 years [3]. Unlike TCAs or MAOIs, viloxazine had relatively low cardiotoxicity, making it safer for elderly patients [3]. Its antidepressant efficacy was primarily attributed to NE reuptake inhibition [2] | [3]. However, no large modern RCTs exist. A review of older clinical trials (1970s–90s) identified at least 37 studies (some unpublished), with target doses ~300–400 mg/day in adults [3]. Across these, viloxazine generally reduced depressive and anxiety symptoms, with the most common side effects being gastrointestinal (nausea, vomiting) and insomnia [3]. It lacked significant anticholinergic or weight gain effects seen with TCAs [2]. In 2002–2008 viloxazine was withdrawn from the European market for commercial reasons (not safety) [4], leaving limited contemporary data. No recent antidepressant trials have been conducted, although its serotonergic activity suggests potential benefit in mood disorders [2].

### 6.2 Off‐Label Uses

Beyond approved ADHD indications and historical depression use, there is minimal evidence for other indications. No controlled trials exist for viloxazine in other psychiatric disorders (e.g., anxiety, OCD, etc.). It is conceivable to consider viloxazine off‐label in comorbid anxiety or refractory depression, given its SNMA profile, but this would be speculative. It may also be tried in ADHD comorbid with mood or tic disorders where stimulants are risky, but formal studies are lacking. Some practitioners might add viloxazine to stimulants to target residual symptoms (Faison et al. 2021 found no PK interaction) [6], but such “adjunct” use is not yet a formal indication. Overall, off‐label prescribing is not supported by robust evidence and would be extrapolated from its mechanism and safety profile.

## 7 Safety and Tolerability

Like all antidepressants, viloxazine carries a Boxed Warning for increased risk of suicidal thoughts and behaviors in children, adolescents, and young adults. In Qelbree trials, suicidal ideation/behavior occurred more often with viloxazine than placebo [1, 7, 8, 10]. The prescribing information emphasizes close monitoring for suicidal thoughts, worsening depression, or unusual behavioral changes [1]. Trials reported no increase in mania beyond expected rates, but noradrenergic drugs can precipitate mania. The label specifically warns that viloxazine “may induce a manic or mixed episode” in bipolar patients [1]. Hence, screening for bipolar disorder prior to initiation is recommended [1]. Other neuropsychiatric effects include irritability and mood swings; in pediatric trials, irritability occurred in ~3%–8% [6]. Hallucinations or psychosis were not commonly reported.

Somnolence (sedation) and fatigue are among the most frequent adverse events (Table 4). In trials, somnolence occurred in 6%–23% of viloxazine‐treated youths vs. < 3% on placebo [1], and 6% of adults vs. 2% on placebo [1] Headache is common (up to ~17% in adults) [1]. Insomnia paradoxically also occurs (8%–9% in youths); the most common adult AE [1]. Patients should be cautioned against driving or operating machinery until they know viloxazine's effect, due to sedation risk [1]. Fatigue and lethargy (5%–12%) are sedating side effects and also appear. The stimulant‐like CNS effects, which include insomnia, decreased appetite, tachycardia, and irritability, are typically mild‐to‐moderate and often resolve or diminish with continued use.

**TABLE 4:** Common treatment‐emergent adverse events (> 5%).

Adverse event	Pediatric (%)	Adult (%)	Placebo (%)	Notes
Somnolence	6%–23%	6%	~2%–3%	Dose‐dependent
Headache	~17%	17%	7%	Often transient
Nausea	10%–12%	12%	3%	Leading cause of discontinuation
Decreased Appetite	~10%	10%	3%	Monitor growth in children
Insomnia	8%–9%	9%	—	Most common AE in adults
Irritability	3%–8%	< 3%	< 1%	Monitor mood changes

Unlike stimulants, viloxazine has a relatively benign cardiovascular profile. As noted, QT prolongation and arrhythmia risks are minimal [6]. However, modest increases in heart rate (∼2–5 bpm) and blood pressure (mean rise ∼1–2 mmHg) were seen in trials [1]. Blood pressure should be assessed before and during treatment [1]. Clinical hypertension occurred in a small number of patients (0.1%–2% above baseline in trials) [6]. Tachycardia was reported in ~4% of viloxazine‐treated adults vs. 1% on placebo [1]. Patients with pre‐existing cardiovascular disease should use viloxazine cautiously.

Nausea and vomiting are common (12% and 4% of adults, 3% each on placebo) [1]. Gastrointestinal symptoms (including dry mouth 10%, constipation 6%) tend to be dose‐related and often improve over time. Decreased appetite is a common adverse effect with viloxazine ER. In adults, the incidence was 10% versus 3% with placebo; in pediatric trials (ages 6–17 years), it ranged from 5% to 8% (overall 7%) versus 0.4% with placebo. Short‐term weight effects were minimal: children gained +0.2 kg on viloxazine versus +1.0 kg on placebo, and adolescents lost −0.2 kg on viloxazine versus gained +1.5 kg on placebo over 6–8 weeks [1], a known effect of NE enhancement. These effects warrant monitoring of weight and growth in children. Diarrhea is not a noted side effect (unlike stimulants).

Rarely, viloxazine can cause altered mood or suicidal ideation in susceptible individuals (hence the boxed warning). Emergence of aggressive behavior or anxiety occurred infrequently (< 2%). Hepatic enzyme elevations were observed in < 1%; no cases of liver failure have been reported. No clinically significant allergy or hematologic toxicities have been identified. Viloxazine lacks the anticholinergic (dry mouth, constipation) and histaminergic (sedation, weight gain) side effects typical of many antidepressants [2]. Overall, viloxazine's tolerability is acceptable: common AEs are manageable, and most trial discontinuations were due to insomnia, nausea, or headache [1, 11] (Table 4 summarizes frequencies).

Viloxazine is contraindicated within 14 days of an MAOI (due to hypertensive crisis risk) [1]. It is also contraindicated with concomitant use of sensitive CYP1A2 substrates or CYP1A2 inhibitors (e.g., clozapine) [1], because viloxazine strongly inhibits CYP1A2, risking toxic levels of substrates. There is no specific contraindication for pregnancy aside from general caution—however, the label advises against use in pregnancy due to potential risks (animal studies indicated maternal harm) [1]. Viloxazine ER is not approved for geriatric use beyond the label stating “no overall differences in efficacy, safety, or PK” in older patients [1], 111 but caution is prudent given reduced clearance in renal impairment.

## 8 Drug Interactions

Viloxazine inhibits several cytochrome P450 enzymes (Table 5). It is a strong inhibitor of CYP1A2, which metabolizes drugs like caffeine, clozapine, olanzapine, and theophylline. Coadministration can greatly increase substrate levels; thus *avoid* co‐use with narrow‐therapeutic‐index CYP1A2 substrates (e.g., tizanidine) [1]. It is a moderate inhibitor of CYP2D6 and CYP3A4 [1]. Consequently, drugs metabolized by these enzymes (certain antidepressants, beta‐blockers, antiarrhythmics) may have elevated plasma levels with viloxazine. Dose reduction or monitoring is advised.

**TABLE 5:** Drug interaction potential of viloxazine.

Interaction type	Enzyme affected	Clinical examples	Recommendation
Strong Inhibition	CYP1A2c	Clozapine, caffeine, tizanidine	Contraindicated
Moderate Inhibition	CYP2D6, CYP3A4	SSRIs, beta‐blockers, warfarin	Dose adjust or monitor
Contraindicated Combination	MAO Inhibitors	Phenelzine, tranylcypromine	Avoid within 14 days
Minimal PK interaction	Lisdexamfetamine	Stimulant ADHD medication	Safe in coadministration
Increased cardiovascular risk	Sympathomimetics	SNRIs, pseudoephedrine	Monitor BP and HR

Combining viloxazine with MAOIs (or within 14 days of discontinuation) is contraindicated [1] (Table 5). Co‐use could precipitate hypertensive crisis or serotonin syndrome due to compounded monoaminergic effects. Similarly, caution is warranted with other serotonergic agents (SSRIs, SNRIs, triptans) as serotonin syndrome is a theoretical risk (though not explicitly reported in trials). If switching from an SSRI/SNRI to viloxazine, allow an appropriate washout period. No pharmacokinetic drug–drug interaction was seen when viloxazine was co‐administered with lisdexamfetamine (a prodrug stimulant) in healthy adults [6]. Thus, viloxazine may be safely combined with stimulants if clinically indicated (though concurrent use was not studied in pivotal ADHD trials). In practice, viloxazine can be added if stimulants alone are insufficient, but this would be off‐label and requires monitoring.

Because viloxazine can increase blood pressure/heart rate slightly, combining with other sympathomimetics (e.g., pseudoephedrine, SNRIs) should be done with caution and monitoring of vital signs. Antihypertensives may be less effective; in some cases, blood pressure control needs adjustment. Viloxazine‐induced appetite suppression can enhance the effects of weight loss drugs (e.g., phentermine). Overall, clinicians should review patients' full medication list for CYP interactions and adjust therapies accordingly.

## 9 Critical Appraisal of Evidence

The evidence for viloxazine is largely drawn from industry‐funded trials sponsored by Supernus (the manufacturer) and published in peer‐reviewed journals. Notably, all pivotal Phase II/III efficacy studies across children, adolescents, and adults were *multicenter*, randomized, double‐blind, placebo‐controlled RCTs conducted at multiple U.S. outpatient sites [7, 8, 10]. Pediatric trials (Nasser et al., Johnson et al.) were well‐designed 6–8 week RCTs with appropriate controls [6, 7]. Across all pivotal trials, participants presented with moderate‐to‐severe ADHD: inclusion criteria required ADHD‐RS‐5 ≥ 28 and CGI‐S ≥ 4 in pediatric and adolescent studies, and AISRS ≥ 26 and CGI‐S ≥ 4 in adult trials, indicating enrollment of subjects with clinically significant symptom severity. These Phase III studies had adequate sample sizes (hundreds of children), randomized double‐blind design, and clear primary endpoints (ADHD‐RS). Risk of bias for efficacy outcomes is low, given placebo control and prespecified analysis. However, sponsorship by the manufacturer is a potential conflict. The data in label and publications were consistent. A limitation is that all pediatric subjects were stimulant‐free (washout) and relatively short (6–8 weeks), so long‐term efficacy and rare AEs cannot be fully assessed.

Adolescent trial also randomized > 290 subjects, but only the 400 mg dose reached significance [10]. This trial appears robust, although statistical significance for 600 mg was missed (possible underpowering for that dose). Overall, the adolescent evidence is moderately strong for 400 mg/day. Adult trial was similarly rigorous (374 subjects) [8]. The effect size on AISRS (LSM difference ~3.8 points) was statistically significant (*p* = 0.004). While this is a modest numerical difference, the responder analyses showed a higher proportion achieving clinical improvement. The trial was well‐controlled, but short duration (6 weeks) and fixed titration. No independent replication has been published, so single‐study limitation applies. One open‐label extension (Childress [11]) suggests sustained efficacy but lacks control.

All pivotal RCTs have authors who are company employees or advisors [3]. While data appear credible, independent replication would strengthen confidence. No head‐to‐head trials with active comparators (stimulants or atomoxetine) have been conducted, except the retrospective chart study (Price) which itself is low‐evidence (unblinded, selection bias) [9]. The long‐term safety data are limited: aside from the 6–12 month open‐label extension (Childress) [11], there are no published multi‐year RCTs.

For FDA‐approved ADHD indications, evidence certainty can be rated moderate. The RCTs provide strong evidence of short‐term symptom reduction, but indirectness (surrogate endpoints) and imprecision (short trials) temper confidence. For historical depression indication, evidence is very low (no modern trials). For safety, the trials were powered to detect common AEs but rare or long‐term risks (e.g., suicide, growth effects) have wide confidence intervals. Thus, we rate ADHD efficacy as moderate‐high, but overall benefit–risk profile requires continued postmarketing surveillance.

## 10 Comparative Efficacy With Other Medications

No large RCT directly compares viloxazine ER with stimulants (methylphenidate or amphetamine) (Table 6). Indirectly, the effect sizes reported (mean ADHD‐RS improvement ~10–15 points) are generally smaller than typical stimulant trials (which often show > 10‐point differences vs. placebo) [8]. Thus, viloxazine's efficacy is likely inferior to stimulants as first‐line agents. Compared to atomoxetine (another nonstimulant NRI), limited data exist. One open‐label study found that patients switching from atomoxetine (mean dose 60 mg) to viloxazine (mean 300 mg) showed greater improvement in ADHD‐RS scores [9]. In this retrospective cohort (*n* = 50, mostly children), mean ADHD‐RS‐5 score dropped more on viloxazine (to 13.9) than on atomoxetine (to 33.1) [9]. Discontinuation rates were lower on viloxazine (4%) versus atomoxetine (36%), mainly due to better tolerability (less GI upset and fatigue on viloxazine) [9]. Although not a randomized trial, this suggests viloxazine may be at least as efficacious and better tolerated than atomoxetine in many patients (Table 6). This aligns with viloxazine's broader mechanism (5‐HT effects) and side effect profile.

**TABLE 6:** Comparative effectiveness vs. atomoxetine and stimulants.

Comparator	Study design	Outcome	Viloxazine vs. comparator
Atomoxetine	Open‐label switch	ADHD‐RS‐5 ↓ from 33.1 to 13.9	Greater efficacy, better tolerability
Stimulants	Indirect	Mean ADHD‐RS change	Slightly less potent than stimulants
Placebo	RCTs	ADHD‐RS‐5 and AISRS	Statistically and clinically superior

In terms of antidepressant comparators, there are no direct data. Given its low serotonergic reuptake potency, viloxazine is not expected to be superior to SSRIs/SNRIs for depression. However, its unique 5‐HT_2C_ agonism could theoretically make it beneficial in cases of anxiety/depression with excessive anxiety (5‐HT_2C_ agonists can have anxiolytic effects) [2]. Clinically, viloxazine lacks direct antihistaminic or anticholinergic receptor blockade (unlike some antidepressants), and sexual dysfunction has not been a prominent signal; however, somnolence and fatigue are common treatment‐emergent events with viloxazine, so any comparative tolerability advantage remains uncertain and unproven without head‐to‐head trials.

Overall, viloxazine offers a new non‐stimulant ADHD option. Its comparative advantage lies in patients who cannot tolerate stimulants or atomoxetine. It may work faster than atomoxetine (signs of effect by 2 weeks) [9], and has a different AE profile. However, if stimulants are tolerated, they remain more potent in symptom control. For depression, viloxazine's role was historically minor and is not positioned against current antidepressants.

## 11 Discussion

Viloxazine ER represents an important addition to ADHD pharmacotherapy, especially for patients who cannot or will not take stimulants. Its SNMA profile offers a distinct mechanism (combined NE/5‐HT modulation) that translates into clinical benefit. The evidence shows significant symptom reduction in ADHD trials [8]. However, the magnitude of effect is modest compared to stimulants, and response rates (e.g., ~45%–55% achieving CGI‐I response in pediatric trials) are lower than stimulants' typical 70%–80%. Safety is reasonable, with somnolence and GI upset as main drawbacks [1]. The boxed warning on suicidality demands vigilance, as with all antidepressant therapies. Given viloxazine's historical antidepressant use and its serotonergic–noradrenergic mechanism (including 5‐HT2C agonism and 5‐HT2B antagonism), it is biologically plausible that viloxazine could benefit patients with ADHD who have comorbid depression. However, no randomized trials have evaluated viloxazine specifically in this comorbid population; therefore, this remains a hypothesis that requires prospective clinical testing. Clinicians should also weigh label warnings (e.g., suicidality) and monitor mood closely if considering such use. All pivotal trials were short‐term (6–8 weeks), so long‐term efficacy, optimal maintenance dose, and durability of response are not fully established. Ongoing open‐label extensions suggest sustained benefit, but placebo‐controlled long‐term data are absent. The evidence is almost exclusively industry‐sponsored; independent trials would help validate findings. Real‐world effectiveness and comparative outcomes (e.g., head‐to‐head vs. atomoxetine or stimulants) are unknown. Off‐label use (e.g., in adult depression or anxiety) has no contemporary support, so such uses remain speculative. Lastly, pediatric data do not include children under 6; safety in that age group is untested.

Viloxazine's arrival underscores interest in nonstimulant ADHD options with novel pharmacology. Future research should focus on: (1) Head‐to‐head trials comparing viloxazine to atomoxetine and stimulants to quantify relative efficacy/tolerability. (2) Long‐term safety studies for growth, cardiovascular effects, and rare AEs over years of use. (3) Biomarker studies to predict which patients benefit most (e.g., genetic polymorphisms affecting metabolism, or neuroimaging correlates). (4) Exploration of other indications, such as relapse prevention in depression or anxiety disorders, leveraging its serotonergic activity. (5) Combination therapies, e.g., viloxazine plus stimulant for partial responders, to assess additive effects.

In practice, viloxazine may be considered after trying first‐line agents. Its lack of abuse potential makes it attractive in adolescents/adults with substance use history. The rapidly growing clinical experience (postmarket surveillance) will clarify its safety (e.g., in pregnancy, among elderly, or with comorbidities). Health economic assessments will determine its place relative to generic alternatives.

In conclusion, viloxazine is a well‐characterized ADHD medication with proven efficacy and a manageable safety profile. Its unique mechanism may also inform future psychiatric drug development (e.g., SNMA class). Clinicians should stay alert to emerging data on viloxazine's long‐term performance and potential new uses.

## 12 Conclusion

Viloxazine ER (Qelbree) is a novel nonstimulant ADHD treatment, approved for children, adolescents, and adults. Preclinical and clinical data show it acts as a moderate norepinephrine reuptake inhibitor with additional serotonergic effects, notably 5‐HT_2C_ agonism. Rigorous trials demonstrate that viloxazine significantly reduces ADHD symptoms versus placebo, with effect sizes smaller than stimulants but comparable to atomoxetine. Its safety profile is distinct: common side effects include somnolence, headache, nausea, and decreased appetite; serious risks (e.g., suicidality) are addressed by warnings. Viloxazine's pharmacokinetics (rapid absorption, 7 h half‐life, CYP metabolism) support once‐daily dosing. Major drug interactions involve CYP1A2 inhibition and MAOI contraindication. The evidence for viloxazine's efficacy in ADHD is moderate‐to‐high quality (multiple RCTs) and has yielded FDA approval. Historical use in depression suggests modest antidepressant effects, but no modern data. Clinicians should weigh viloxazine as an option for patients requiring nonstimulant treatment. Further research is needed to fully establish viloxazine's long‐term outcomes, comparative place among therapies, and potential broader psychiatric applications.

## Conflicts of Interest

The authors declare no conflicts of interest.

## Data Availability

Data sharing not applicable to this article as no datasets were generated or analysed during the current study.
